# Sleep Quality and Chronotype Differences between Elite Athletes and Non-Athlete Controls

**DOI:** 10.3390/clockssleep1010002

**Published:** 2018-09-05

**Authors:** Amy M. Bender, Hans P. A. Van Dongen, Charles H. Samuels

**Affiliations:** 1 Faculty of Kinesiology, University of Calgary, Calgary, AB T2N 1N4, Canada; 2 Sleep and Performance Research Center, Washington State University, Spokane, WA 99202, USA; 3 Elson S. Floyd College of Medicine, Washington State University, Spokane, WA 99202, USA; 4 Centre for Sleep and Human Performance, Calgary, AB T2X 3V4, Canada

**Keywords:** sleep disturbance, morningness, eveningness, circadian misalignment

## Abstract

Previous research has found that elite athletes have insufficient sleep, yet the specific kinds of sleep disturbances occurring as compared to a control group are limited. Here we compare the subjective sleep quality and chronotype of elite athletes to a control group of non-athlete good sleepers. Sixty-three winter Canadian National Team athletes (mean age 26.0 ± 0.0; 32% females) completed the Pittsburgh Sleep Quality Index (PSQI) and the Athlete Morningness Eveningness Scale. They were compared to 83 healthy, non-athlete, good-sleeper controls (aged 27.3 ± 3.7; 51% females) who completed the PSQI and the Composite Scale of Morningness. The elite athletes reported poorer sleep quality (PSQI global score 5.0 ± 2.6) relative to the controls (PSQI global score 2.6 ± 1.3), despite there being no group difference in self-reported sleep duration (athletes 8.1 ± 1.0 h; controls 8.0 ± 0.7 h). Further, athletes’ chronotype distribution showed a greater skew toward morningness, despite there being no group differences in self-reported usual bedtime and wake time. These results suggest that a misalignment of sleep times with circadian preference could contribute to poorer sleep quality in elite athletes.

## 1. Introduction

Insufficient sleep has been shown to cause metabolic deficiencies [1,2], reduce muscle strength [3], shorten time to exhaustion during exercise [4], alter perceived exertion during exercise [5,6], and increase lapses of attention and reaction times [7,8]. All these effects can be detrimental to athletic performance and underline the importance of elite athletes obtaining sufficient sleep. Sleep sufficiency depends on components of quality, quantity, and timing of the sleep window [9]. Research has shown benefits of optimized sleep for athletic performance [10,11,12]. Even so, knowledge regarding the sleep behaviors of athletes is limited [13,14]. 

Previous research has found that elite athletes have poor sleep quality [9,15,16,17]. For example, more than 55% of athletes in the Bobsleigh Canada Skeleton team were flagged as being poor sleepers [9]. Poor sleep was also observed in 50% of a sample of 24 professional ballet dancers [15], 50% of 175 elite rugby and cricket athletes [16], and 25% of 107 professional Finnish hockey athletes [18]. Despite such evidence for a high prevalence of poor sleep in elite athletes, little is known about what kinds of sleep disturbances occur. 

Another important aspect of sleep in athletes is sleep quantity, which may be operationally defined as the sum of all sleep across a 24-h day including both nighttime and daytime sleep [19]. The recommended amount of sleep for adults is between 7 h and 9 h per day [20]. According to the available evidence, many athletes may not meet the minimum sleep quantity recommendation [21,22,23]. A study in 47 Olympic athletes from various sports documented an average of 6.9 h of sleep across four nights, as measured objectively by actigraphy [21]. A similar amount of sleep was found in 124 elite Australian athletes from both individual and team sports, with team athletes averaging 6.8 h and athletes from individual sports averaging 6.5 h of sleep per night [22]. Research in Australian Olympic swimmers found that during early morning training days, athletes obtained just 5.4 h of sleep on average, compared to 7.1 h of sleep on rest days [23]. Competing at night was associated with higher salivary cortisol and decreased quantity and quality of sleep [24]. An intervention study in collegiate basketball athletes documented that when sleep was extended from less than 7 h per night to more than 8 h per night, mood, sleepiness, reaction time, and shooting percentage were improved [10]. 

Misalignment of the scheduled sleep opportunity with the circadian rhythm of the biological clock may contribute to insufficient quality and quantity of sleep [25]. Such misalignment is often present in athletes who travel rapidly across time zones [26,27], but may also occur systemically in morning-type or evening-type athletes who are forced to train or compete outside their biological, endogenous sleep window. There are both biological and behavioral aspects to circadian phase preference or “chronotype” [28,29]. The biological and behavioral determinants of chronotype are not well understood in athletes, yet they can have a measurable impact on training and athletic performance [30]. A study in young, healthy, non-athletes (ages 27 ± 7 years) classified 14% of individuals as morning types [31]. In athletes, it appears there is a higher prevalence of morning types than in the general population [9,32,33,34]. Kunorozva et al. (2012) found that 37% of 532 elite and sub-elite cyclists, runners, and Ironman triathletes were morning types, who preferred training in the morning [32]. Morning preference was also found in 37% of 21 Bobsleigh Canada Skeleton athletes [9], 72% of 27 Brazilian Paralympic athletics athletes [33], and 29% of 114 elite cricket, cycling, hockey, soccer, and triathlon athletes [34]. 

A recent, systematic review found a high prevalence of poor sleep quality in elite athletes [13]. However, the overall quality of evidence was found to be poor, with few controlled comparisons between elite athletes and non-athlete controls. Of the 37 studies included in the review, only three (8%) incorporated a control-group study design. In a comparison between 46 British Olympic athletes and 20 non-athletes based on actigraphic recordings of sleep, Leeder and co-authors documented that the athletes exhibited lower sleep efficiency due to longer sleep latencies and more wake after sleep onset [21]. Based on assessments using the Pittsburgh Sleep Quality Index (PSQI), Tsunoda and colleagues reported that Japanese wheelchair basketball athletes had poorer sleep quality and sleep efficiency than able-bodied non-athletes [35]. In contrast, a study by Driller and colleagues observed poorer PSQI global scores in 322 non-athletes compared to 242 athletes [36]. A study by Lastella and co-authors, based on self-report data, did not find any significant differences in sleep between 114 elite Australian athletes and 82 age-matched controls [34]. 

Given these mixed results, more research is needed to determine if athletes have poorer sleep quality when compared to controls, and what kinds of sleep disturbances may be observed. The current study therefore examined the subjective assessment of sleep and circadian phase preference in athletes and a control group of good sleepers in the same age range. We hypothesized that athletes have a higher prevalence of morningness and poorer sleep quality than non-athletes. 

## 2. Results

### 2.1. Participants

We studied *n* = 63 elite athletes with a mean age (±SD) of 26.0 (±4.0) years, and *n* = 83 healthy controls with a mean age (±SD) of 27.3 (±4.7) years. There was no significant age difference between athletes and controls (t_144_ = 1.71, *p* = 0.090). Thirty-two percent (20/63) of the athletes were female, compared to 51% of the controls (42/83).

### 2.2. Sleep

**Figure 1:**
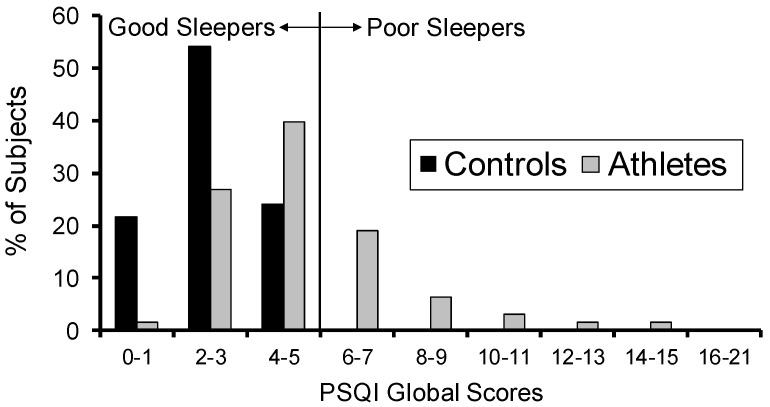
Distribution of Pittsburgh Sleep Quality Index (PSQI) global sleep quality scores. Those with PSQI global scores ≤ 5 are classified as good sleepers; those with global scores > 5 are classified as poor sleepers.

Figure 1 shows the distribution of PSQI global scores in the elite athletes and the non-athlete controls. Compared to subjects in the control group (who were screened to be good sleepers), the elite athletes had worse sleep quality scores, with 32% being categorized as poor sleepers (defined as PSQI scores > 5). There was a significant difference between the two groups (t_85_ = 6.82, *p* < 0.001), with the athletes exhibiting poorer sleep quality scores (mean ± SD: 5.0 ± 2.6) compared to the controls (2.6 ± 1.3).

For the PSQI component score of sleep quality, 10% of athletes rated their sleep as fairly bad, compared to none of the controls. There was a significant difference between the two groups for this component (Z = 3.82, *p* < 0.001). 

For the PSQI component of sleep latency, 37% of athletes reported having trouble falling asleep at least once a week, compared to 4% of the controls. There was a significant difference between the two groups (Z = 5.05, *p* < 0.001). 

**Figure 2:**
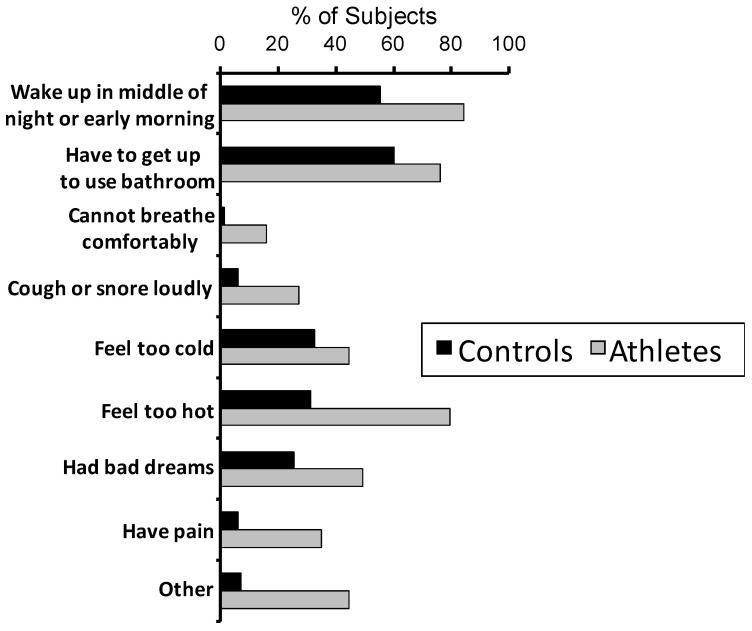
Distribution of reasons for trouble sleeping during the past month for the sleep disturbances component of the PSQI.

For the PSQI component of sleep disturbances, the athletes consistently exceeded the controls on the frequency of trouble sleeping for nine possible reasons, as shown in Figure 2. There was a significant difference between the two groups (Z = 5.75, *p* < 0.001).

For the PSQI component of daytime dysfunction, the elite athletes showed less trouble staying awake but more difficulty having enough enthusiasm to get things done. There was a significant difference between the two groups (Z = 6.22, *p* < 0.001). 

For the PSQI component of medication use, 16% of the athletes reported taking medicine that was prescribed or over the counter to help them sleep, compared to 1% of the controls. There was a significant difference between the two groups (Z = 3.32, *p* < 0.001). 

There were no significant group differences for the remaining PSQI components of sleep duration (Z = 0.44, *p* = 0.663) and sleep efficiency (Z = 0.01, *p* = 0.988). 

There were no significant differences between the two groups in self-reported usual bedtime, sleep duration, and getting up time. However, for self-reported sleep latency, there was a significant difference between the groups (t_92_ = 2.47, *p* = 0.015) (see Table 1).

### 2.3. Chronotype

**Figure 3:**
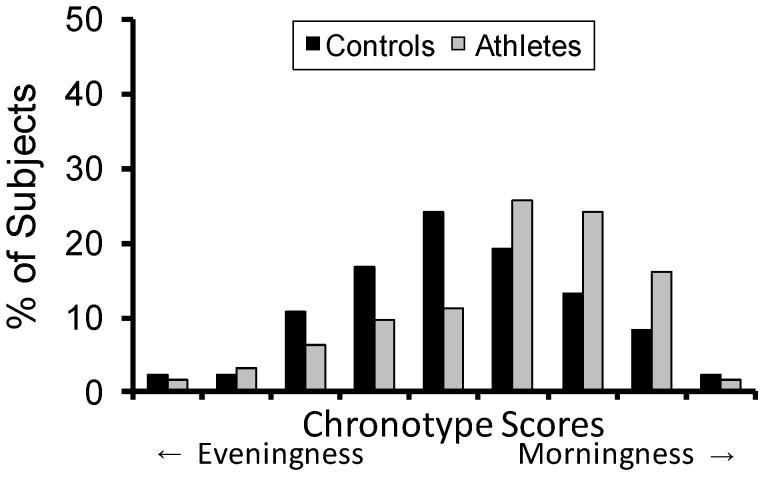
Distribution of chronotype scores. Questionnaire scores were binned into nine equal intervals prior to analysis.

Chronotype scores were distributed differently between the elite athletes and the non-athlete controls (Z = 2.48, *p* = 0.013). As shown in Figure 3, there was a greater prevalence of morningness in the athletes. 

In the athletes, chronotype was significantly correlated with sleep quality (*r* = −0.35, *p* = 0.005), such that greater eveningness (lower chronotype scores) was associated with poorer sleep quality (higher PSQI scores). For the non-athlete controls, chronotype was not significantly correlated with sleep quality (*r* = −0.17, *p* = 0.11).

## 3. Discussion

### 3.1. Study Findings

The main objective of this study was to investigate subjective sleep quality and chronotype differences between a group of elite athletes and a control group of non-athlete, good sleepers. Previous research has reported poor sleep in elite athletes, but with limited comparisons to controls [21,34,35]. Here, we found that elite athletes, as compared to controls, experienced poorer sleep, as evidenced by degraded sleep quality, difficulty falling asleep, more sleep disturbances, greater sleep medication use, and daytime dysfunction. As this was found in the absence of a significant group difference in self-reported sleep duration, this suggests that the elite athletes experienced problems primarily with the subjective quality rather than the subjective quantity of their sleep.

In line with previous research [9,32,33,34] we observed a relatively high prevalence of morningness among the elite athletes. It is unclear whether this was a result of external pressures producing social jet lag [37] and/or endogenous neurobiological differences in circadian phase [38]. Lastella and colleagues found a higher prevalence of morning and intermediate chronotypes in triathlon, a sport that typically has early morning training sessions [34]. The authors concluded that athletes may self-select into sports with training times aligned with their chronotype, yielding a greater likelihood to excel at that sport. Laborde and co-workers reported a similar finding in recreational athletes, as those who were evening types tended to participate in sport activities later in the day [39]. In the current study, we did not collect information on the actual training times of the athletes, but the self-reported usual bedtimes and wake times were similar to those found in the controls. This suggests a misalignment between preferred sleep times and actual sleep times in the athletes, which may provide an explanation for the poor sleep quality seen in this sample. 

In Australian Olympic swimmers, Sargent and colleagues found a 2.5-h discrepancy between bedtimes and a 4-h discrepancy between wake times on training days versus rest days, resulting in a 1.6-h gain in sleep duration on rest days [23]. A similar pattern was found in young elite Australian judo athletes, who woke up 1.5 h later and gained 45 min in sleep duration when morning training times were delayed from 06:30 to 09:30 [40]. Although these earlier studies did not assess sleep quality or use a control group study design, their results are consistent with the idea that misalignment between preferred and actual sleep times may degrade elite athletes’ sleep. This possibility is further corroborated by the present finding that athletes with greater tendency toward eveningness reported poorer sleep quality. It would have been informative to know whether those evening types with the poorest sleep quality were required to train early in the morning. Further research is needed to investigate whether delaying training start times could result in long-term improvements in the sleep of elite athletes, and whether that would be associated with improvements in training, recovery, and performance. 

Given that elite athletes are subjected to physically and cognitively taxing activities, we should also consider the possibility that they may have a greater sleep need than non-athletes. Little is known about the sleep requirements of elite athletes [14], but the results of sleep extension studies are telling. For example, athletic performance in basketball and tennis athletes was found to be improved when sleep duration was extended [10,11], albeit without comparison to a control group. This intriguing finding suggests that even if sleep times were to be aligned with athletes’ circadian preferences and this were to result in improved sleep, optimal athletic performance might not be achieved unless sleep duration is extended as well. 

### 3.2. Limitations

The tools we used to assess sleep quality (i.e., the PSQI) and chronotype (i.e., the Athlete Morningness Eveningness Scale (AMES) and Composite Scale of Morningness (CSM)) were self-report instruments (questionnaires described below). Furthermore, subjects in the non-athlete control group were screened to have PSQI scores ≤ 5, thereby representing a sample of good sleepers that is not representative of the population at large. Therefore, although our data raise potential concerns about the sleep of elite athletes, we must be careful not to interpret the results as definitive evidence that poor sleep quality is more prevalent in elite athletes than in non-athletes. In addition, we used different questionnaires to determine chronotype in the athletes (i.e., the AMES) versus the controls (i.e., the CSM). We applied a transformation to the data to make sure they were on the same scale prior to analysis. Nonetheless, we cannot be certain that the psychometric properties of the (transformed) chronotype scores were equivalent between the two groups. Although our data suggest that morningness is more prevalent among athletes than among controls, we must exercise caution in the interpretation of that finding. 

Using objective measurement tools would have helped to overcome these limitations. While sleep quality is inherently subjective, objectively measurable correlates of poor sleep quality have been proposed, including frequency of arousals [41] and sleep discontinuity [42]. Likewise, while circadian phase preference is inherently subjective, the timing of the circadian pacemaker (biological clock) can be measured objectively based on the timing of the dim light melatonin onset [43] or, more recently, through transcriptome-based biomarkers in blood [44]. Thus, given sufficient resources, future research should be able to address questions about athletes’ sleep disturbances and the underlying causes more definitively.

## 4. Materials and Methods

### 4.1. Participants

*Elite Athletes*: We recruited 63 national and Olympic winter team athletes between the ages of 22 and 38 from the Canadian Sport Centre Calgary. The athletes had been at their current national team level for an average of 5.8 years (±3.5 SD) and represented 11 different sports, including bobsleigh (*n* = 12), cross country skiing (*n* = 11), long track speed skating (*n* = 9), skeleton (*n* = 7), luge (*n* = 6), biathlon (*n* = 6), short track speed skating (*n* = 5), ski cross (*n* = 4), curling (*n* = 2), and snowboarding (*n* = 1).

*Controls*: Data from the elite athletes were compared to 83 healthy, non-athlete controls between the ages of 22 and 38, taken from three studies previously completed at Washington State University’s Sleep and Performance Research Center. Specifically, there were 15 subjects taken from study 1, 48 subjects taken from study 2, and 20 subjects taken from study 5 as published previously [45]. Controls were screened to be physically and psychologically healthy as verified with medical history and physical exam, with no current medical or drug treatment (except oral contraceptives). They were free of alcohol and drugs, not current smokers, and had no clinically relevant history of alcohol and drug abuse. They were also screened to be good sleepers and free from sleep or circadian rhythm disorders as verified with questionnaires and polysomnography; no shift work in the past 3 months (studies 2 and 5); and no travel to other time zones in the past month (studies 2 and 5). They had a self-reported sleep duration of 6–10 h per night (for study 1: 7–9 h) and a habitual wake time between 06:00 and 09:00 (for study 1: between 06:30 and 08:30). For studies 1 and 5, subjects were excluded for being an extreme morning or evening type by self-report. 

The study protocols were approved by the Conjoint Health Research Ethics Board at the University of Calgary (elite athletes) and the Institutional Review Boards of Washington State University and the University of Pennsylvania (controls). All participants gave written informed consent. 

### 4.2. Questionnaires

Sleep Quality: The Pittsburgh Sleep Quality Index (PSQI) is a self-rated, 19-item, validated questionnaire used to evaluate sleep quality and sleep disturbances over the past month [46]. The items are used to generate seven component scores: sleep quality, sleep latency, sleep duration, sleep efficiency, sleep disturbances, use of sleeping medication, and daytime dysfunction. The component scores are also compiled to create an overall sleep quality global score, with a range of 0–21. A PSQI global score cut-off of 5 has been established to distinguish good sleepers (≤5) from poor sleepers (>5) [46]. Subjects in the control group were screened to be good sleepers, having PSQI global score ≤ 5.

The PSQI also asks subjects to report their usual bedtime, sleep latency, getting up time, and sleep duration during the past month. These items were considered separately from the PSQI global and component scores. 

Chronotype: The Athlete Morningness Eveningness Scale (AMES), which is based on the classical Horne-Östberg morningness-eveningness questionnaire [47], is a 4-item questionnaire designed to assess chronotype in athletes based on timing of tiredness, self-identification of being a morning or evening type, and preferred training and competition times [48]. The Composite Scale of Morningness (CSM), which is based on two morningness–eveningness questionnaires [49], is a 13-item scale with good psychometric properties designed to assess chronotype in young adults [50].

### 4.3. Statistical Analyses

Statistical analyses were performed using SAS 9.4 (SAS Institute, Cary, NC, USA). The type I error threshold was set to α = 0.05. Two-sample *t*-tests were used to examine differences between elite athletes and controls for the following variables: age, PSQI global scores, bedtime, getting up time, sleep latency, and sleep duration. Satterthwaite’s approximation for degrees of freedom was applied if a variance ratio test for equality of variances indicated unequal variances between groups. Wilcoxon rank-sum tests were used to examine differences between elite athletes and controls for the seven PSQI component scores and chronotype. 

To ensure comparability of the two different chronotype questionnaires, scores from each of the questionnaires were binned into nine equal intervals prior to analysis. A Pearson product-moment correlation coefficient was computed to evaluate the relationship between PSQI global scores and chronotype scores in the elite athletes and controls. Figures are presented as percentage of subjects to control for the differences in sample size between the two groups.

## 5. Conclusions

Interest in sleep as a passive recovery tool for elite athletes and the role of circadian rhythmicity in recovery and performance has been growing in the sport science community. In the present study, we investigated sleep quality and chronotype in elite athletes, and compared them to a group of non-athlete controls. We hypothesized that the athletes would have a higher prevalence of morningness and poorer sleep quality than the non-athletes. Consistent with our hypothesis, we found that the athletes reported poorer sleep quality and a greater prevalence of morningness in the chronotype distribution. However, there were no group differences in self-reported usual bedtime and wake time. This may imply a misalignment of sleep times with circadian preference, which could contribute to the observed, poorer sleep quality in the athletes. Our findings provide self-report evidence to begin exploring specific sleep and circadian issues in elite athletes, so that any future changes in training and recovery regimens are based on good evidence.

## Figures and Tables

**Table 1:** Self-reported sleep parameters.

	Athletes	Controls			
	Mean ± SD	Mean ± SD	*t*	df	*p*
Bedtime (HH:MM ± min)	22:56 ± 57	22:45 ± 50	1.15	144	0.25
Sleep latency (min)	21.5 ± 16.9	15.2 ± 12.9	2.47	92	0.015 *
Sleep duration (h)	8.1 ± 1.0	8.0 ± 0.7	0.23	108	0.82
Getting up time (HH:MM ± min)	07:44 ± 67	07:32 ± 55	1.17	144	0.24

SD = standard deviation; df = degrees of freedom; HH:MM = time in hours:minutes. * *p* < 0.05.

## Abbreviations

PSQI	Pittsburgh Sleep Quality Index
AMES	Athlete Morningness Eveningness Scale
CSM	Composite Scale of Morningness
